# Enhanced expression of β cell Ca_V_3.1 channels impairs insulin release and glucose homeostasis

**DOI:** 10.1073/pnas.1908691117

**Published:** 2019-12-23

**Authors:** Jia Yu, Yue Shi, Kaixuan Zhao, Guang Yang, Lina Yu, Yuxin Li, Eva-Marie Andersson, Carina Ämmälä, Shao-Nian Yang, Per-Olof Berggren

**Affiliations:** ^a^The Rolf Luft Research Center for Diabetes and Endocrinology, Karolinska Institutet, SE-171 76 Stockholm, Sweden;; ^b^Institute of Pharmacology, Jilin Academy of Traditional Chinese Medicine, Changchun 130021, China;; ^c^National Engineering Laboratory for Druggable Gene and Protein Screening, Northeast Normal University, Changchun 130024, China;; ^d^Bioscience, Research and Early Development, Cardiovascular, Renal and Metabolism, BioPharmaceuticals R&D, AstraZeneca, 431 38 Mölndal, Gothenburg, Sweden;; ^e^Department of Endocrinology and Metabolism, West China Hospital of Sichuan University, Chengdu 610041, China

**Keywords:** calcium channel, diabetes, exocytotic proteins, forkhead box O transcription factor, insulin secretion

## Abstract

Voltage-gated calcium 3.1 (Ca_V_3.1) channels are absent in healthy mouse β cells and mediate minor T-type Ca^2+^ currents in healthy rat and human β cells but become evident under diabetic conditions. Whether more active Ca_V_3.1 channels affect insulin secretion and glucose homeostasis remains enigmatic. We addressed this question by enhancing de novo expression of β cell Ca_V_3.1 channels and exploring the consequent impacts on dynamic insulin secretion and glucose homeostasis as well as underlying molecular mechanisms with a series of in vitro and in vivo approaches. We now demonstrate that a recombinant adenovirus encoding enhanced green fluorescent protein–Ca_V_3.1 subunit (Ad-EGFP-Ca_V_3.1) efficiently transduced rat and human islets as well as dispersed islet cells. The resulting Ca_V_3.1 channels conducted typical T-type Ca^2+^ currents, leading to an enhanced basal cytosolic-free Ca^2+^ concentration ([Ca^2+^]_i_). Ad-EGFP-Ca_V_3.1-transduced islets released significantly less insulin under both the basal and first phases following glucose stimulation and could no longer normalize hyperglycemia in recipient rats rendered diabetic by streptozotocin treatment. Furthermore, Ad-EGFP-Ca_V_3.1 transduction reduced phosphorylated FoxO1 in the cytoplasm of INS-1E cells, elevated FoxO1 nuclear retention, and decreased syntaxin 1A, SNAP-25, and synaptotagmin III. These effects were prevented by inhibiting Ca_V_3.1 channels or the Ca^2+^-dependent phosphatase calcineurin. Enhanced expression of β cell Ca_V_3.1 channels therefore impairs insulin release and glucose homeostasis by means of initial excessive Ca^2+^ influx, subsequent activation of calcineurin, consequent dephosphorylation and nuclear retention of FoxO1, and eventual FoxO1-mediated down-regulation of β cell exocytotic proteins. The present work thus suggests an elevated expression of Ca_V_3.1 channels plays a significant role in diabetes pathogenesis.

## Significance

We reveal that increased expression of Ca_V_3.1 channels in rat islets selectively impairs first-phase glucose-stimulated insulin secretion. This deterioration is recapitulated in human islets. Its causal role in diabetes development is clearly manifested in an in vivo diabetic model. Mechanistically, this is due to reduction of phosphorylated FoxO1 in the cytoplasm, elevated FoxO1 nuclear retention, and decreased syntaxin 1A, SNAP-25, and synaptotagmin III in a Ca_V_3.1 channel- and calcineurin-dependent manner. Our findings suggest that elevated expression of Ca_V_3.1 channels in pancreatic islets drives FoxO1-mediated down-regulation of exocytotic proteins resulting in the diabetic phenotypes of impaired insulin secretion and aberrant glucose homeostasis. This causal connection pinpoints β cell Ca_V_3.1 channels as a potential druggable target for antidiabetes therapy.

Multiple types of voltage-gated calcium (Ca_V_) channels, including Ca_V_3.1, operate in the pancreatic β cell mediating Ca^2+^ influx in response to membrane depolarization evoked by increased blood glucose (1–7). Ca^2+^ influx through all types of Ca_V_ channels and Ca^2+^ mobilization mediated by intracellular Ca^2+^-release channels in the β cell regulate glucose-stimulated insulin secretion (1, 5–8). In this context, β cell Ca_V_1 channels serve as a predominant player compared to other types of β cell Ca_V_ channels due to an optimal elevation in cytosolic free Ca^2+^ concentration ([Ca^2+^]_i_) within exocytotic sites (1, 5–7). This optimally elevated [Ca^2+^]_i_ allosterically initiates direct interactions between exocytotic proteins such as VAMP-2, synaptotagmins III and VII in the insulin granule membrane, and syntaxin 1A and SNAP-25 in the plasma membrane (5–7, 9). These interactions trigger fusion of insulin granules with the plasma membrane to form fusion pores for insulin cargo release in the first phase of glucose stimulation (5–7, 9). It is most likely that Ca^2+^ influx mediated by other types of β cell Ca_V_ channels mildly raises [Ca^2+^]_i_ in regions which do not overlap well with exocytotic sites (5–7). This appears to facilitate trafficking of insulin granules to the readily releasable pool to be involved in late-phase but not early-phase glucose-stimulated insulin secretion (5–7). Although it has been suggested that physiological Ca^2+^ influx through Ca_V_3 channels participates in glucose-stimulated insulin secretion, the underlying mechanisms are not known (6, 7, 10, 11).

The Ca_V_3.1 channel is absent in healthy mouse β cells, whereas it resides in healthy rat and human β cells to conduct T-type Ca^2+^ currents, accounting for only a minor proportion of total Ca_V_ currents (6, 7, 10, 12, 13). T-type Ca^2+^ currents appear in diabetic mouse β cells and are significantly increased in diabetic rat β cells (14, 15). Despite little understanding of the physiological role of the Ca_V_3.1 channel in the β cell, there is a possibility that this Ca^2+^ channel affects expression of exocytotic proteins under diabetic conditions (6, 7). Ca^2+^-dependent protein phosphatase calcineurin (CaN) undergoes activation when [Ca^2+^]_i_ increases (16). Activated CaN may suppress the expression of exocytotic proteins through dephosphorylation of FoxO1 (17–19). Systemic administration of Ca_V_3 channel blockers significantly ameliorates hyperglycemia in diabetic mice (20). However, the causal role and transcriptomic impact of a pathological elevation of β cell Ca_V_3.1 channels in the development of diabetes is not known. We hypothesized that excessive Ca^2+^ influx through up-regulated Ca_V_3.1 channels drives β cell dedifferentiation by constitutively hyperactivating CaN and downstream FoxO1 signaling. This is likely to result in impaired expression of β cell exocytotic proteins, disturbed insulin secretion, aberrant glucose homeostasis, and consequent diabetes. Our results verify that the hypothesized signaling pathway indeed operates in β cells up-expressing Ca_V_3.1 channels, suggesting their important role in the pathogenesis of diabetes. This also means that Ca_V_3.1 channels may serve as potential druggable targets in the treatment of diabetes.

## Results and Discussion

### Ad-EGFP-Ca_V_3.1 Efficiently Transduces COS-7 and Rat Islet Cells, and Expressed EGFP-Ca_V_3.1 Conducts Typical T-Type Ca^2+^ Currents.

Recombinant adenovirus vectors have been verified to efficiently transduce islets (21). To elevate Ca_V_3.1 channel expression in rat islets, we constructed recombinant adenoviruses carrying either enhanced green fluorescent protein (Ad-EGFP) or the EGFP-Ca_V_3.1 subunit (Ad-EGFP-Ca_V_3.1) and characterized them in COS-7 cells. Ad-EGFP (*SI Appendix*, Fig. S1 *A*, *Top*) and Ad-EGFP-Ca_V_3.1 (*SI Appendix*, Fig. S1 *A*, *Bottom*) were efficiently expressed in COS-7 cells. Ad-EGFP-Ca_V_3.1-positive cells displayed typical T-type Ca^2+^ currents characterized by tiny unitary conductance and fast inactivation (*SI Appendix*, Fig. S1 *B*, *Right*), whereas Ad-EGFP-positive cells showed no Ca^2+^ currents (*SI Appendix*, Fig. S1 *B*, *Left*). Furthermore, the former cells expressed genuine whole-cell T-type Ca^2+^ currents, which were transiently activated at more negative membrane potentials (*SI Appendix*, Fig. S1*C*, third and fourth panels), but the latter cells did not (*SI Appendix*, Fig. S1*C*, second panel). The effective expression and adequate functionality of Ad-EGFP-Ca_V_3.1 in COS-7 cells offer a promising basis for elevation of Ca_V_3.1 channels in rat islets.

**Fig. 1.**
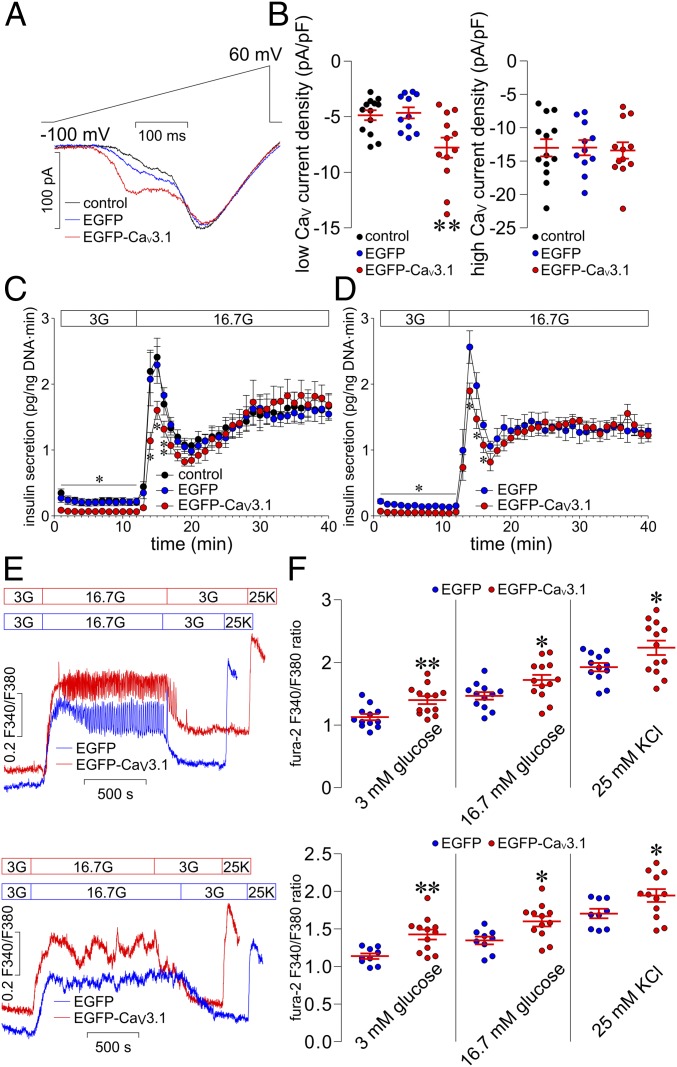
Effects of Ad-EGFP-Ca_V_3.1 transduction on whole-cell Ca_V_ currents in dispersed islet cells and on glucose-stimulated insulin secretion and [Ca^2+^]_i_ in islets. (*A*) Sample whole-cell Ca_V_ current traces from a control rat islet cell and a rat islet cell transduced with Ad-EGFP or Ad-EGFP-Ca_V_3.1. (*B*) Average Ca^2+^ current density–voltage relationships in control (*n* = 13), Ad-EGFP (*n* = 11), and Ad-EGFP-Ca_V_3.1 (*n* = 12) groups. **P < 0.01 vs. control and Ad-EGFP. (*C* and *D*) Dynamic insulin secretion from control, Ad-EGFP-transduced, and Ad-EGFP-Ca_V_3.1-transduced rat (*C*) and human (*D*) islets perifused with 3 mM glucose (3G) followed by 16.7 mM glucose (16.7G). **P < 0.01 and *P < 0.05 vs. control and Ad-EGFP. Control rat islets (*n* = 13), Ad-EGFP-transduced rat islets (*n* = 10), Ad-EGFP-Ca_V_3.1-transduced rat islets (*n* = 10), Ad-EGFP-transduced human islets (*n* = 6), and Ad-EGFP-Ca_V_3.1-transduced human islets (*n* = 6). (*E*) Sample [Ca^2+^]_i_ traces registered in Ad-EGFP- and Ad-EGFP-Ca_V_3.1-transduced rat (*Upper*) and human (*Lower*) islets following perifusion with 3 mM (3G) and 16.7 mM glucose (16.7 G) as well as 25 mM KCl (25K). (*F*) Quantitative analysis of average fura-2 F340/F380 ratios during exposure to 3 mM glucose and stimulation with 16.7 mM glucose and peak fura-2 F340/F380 ratios in response to 25 mM KCl in Ad-EGFP-transduced (*n* = 12) and Ad-EGFP-Ca_V_3.1-transduced (*n* = 13) rat islets (*Upper*) and human islets (*n* = 9 for Ad-EGFP and *n* = 12 for Ad-EGFP-Ca_V_3.1) (*Lower*). **P < 0.01 and *P < 0.05 vs. Ad-EGFP.

We characterized expression and functionality of Ad-EGFP-Ca_V_3.1 in dispersed rat islet cells using the same experimental approaches as employed in the above experiments. We observed that both Ad-EGFP-infected (*SI Appendix*, Fig. S2, *Top*) and Ad-EGFP-Ca_V_3.1-infected islet cells (*SI Appendix*, Fig. S2, *Bottom*) emitted intense EGFP fluorescence. This substantiates that these recombinant adenoviral vectors can effectively express the EGFP and Ca_V_3.1 genes in these cells. We also visualized 2 inward current components, peaking at about −45 and −5 mV, in control cells and cells transduced with Ad-EGFP- or Ad-EGFP-Ca_V_3.1 (Fig. 1*A*). The first component, i.e., low Ca_V_ currents, was significantly greater in Ad-EGFP-Ca_V_3.1-treated cells than in control cells or cells infected with Ad-EGFP (Fig. 1 *A* and *B*). However, there was no obvious difference in the second component, namely, high Ca_V_ currents, between these 3 types of cells (Fig. 1 *A* and *B*). The data reveal that Ad-EGFP-Ca_V_3.1 can satisfactorily transduce dispersed rat islet cells and compel these cells to additionally express functional Ca_V_3.1 channels, thereby enhancing T-type Ca^2+^ currents.

It should be pointed out that we rarely visualized EGFP fluorescence in the plasma membrane of EGFP-Ca_V_3.1-positive COS-7 (*SI Appendix*, Fig. S1*A*) and islet cells (*SI Appendix*, Fig. S2). Nevertheless, we could exclusively detect whole-cell T-type Ca^2+^ currents in these cells. This is due to the fact that a limited number of EGFP-Ca_V_3.1 channels scatter throughout the plasma membrane and have a low probability of appearing in the tiny proportion of the plasma membrane in the focal plane of the confocal microscope.

### Ad-EGFP-Ca_V_3.1-Transduced Islets Display Impairments in Both Basal and First-Phase Glucose-Stimulated Insulin Secretion and Elevation in Basal [Ca^2+^]_i_.

Successful transduction of COS-7 and dispersed islet cells does not necessarily mean that the same thing should happen in intact islets, which are more difficult to transduce (22). Therefore, we evaluated the efficiency of infection of rat and human islets with Ad-EGFP-Ca_V_3.1. Both Ad-EGFP (*SI Appendix*, Fig. S3 *A* and *B*, *Top* 2 panels) and Ad-EGFP-Ca_V_3.1 (*SI Appendix*, Fig. S3 *A* and *B*, *Bottom* 2 panels) satisfactorily infected islet cells within intact islets that emit intense EGFP fluorescence. This means that the Ca_V_3.1 gene in Ad-EGFP-Ca_V_3.1 can be efficiently expressed in cells within intact islets. Pancreatic β cells are specialized for glucose-stimulated insulin secretion (6, 7, 12). The importance of any molecular constituent of the β cell should be reflected upon by the extent and severity of impairment in glucose-stimulated insulin secretion when altered. To explore the importance of Ca_V_3.1 channels in β cells, we analyzed dynamic glucose-stimulated insulin secretion from rat and human islets transduced with Ad-EGFP-Ca_V_3.1. Ad-EGFP-Ca_V_3.1-transduced islets released significantly less insulin during perifusion with 3 mM glucose compared to control islets and islets treated with Ad-EGFP (Fig. 1 *C* and *D*). Importantly, Ad-EGFP-Ca_V_3.1-transformed islets displayed a selective impairment in first-phase glucose-stimulated insulin secretion with intact second-phase insulin response when exposed to 16.7 mM glucose (Fig. 1 *C* and *D*). Moreover, elevated expression of β cell Ca_V_3.1 channels impaired glucose-stimulated insulin secretion not only from rat but also human islets. These data suggest that the pathologically enhanced expression of β cell Ca_V_3.1 channels serves as a causal factor for defective insulin secretion from human and rodent islets, regardless of obvious interspecies differences in expression of Ca_V_3.1 channels in healthy β cells (6, 7, 10, 12, 13). This is well supported by the findings of the appearance of T-type Ca^2+^ currents in diabetic mouse β cells, elevation of these Ca^2+^ currents in diabetic rat β cells, and, in particular, amelioration of hyperglycemia by systemic inhibition of these Ca^2+^ currents in diabetic mice (6, 7, 12, 14, 15, 20). Taken together, the findings indicate the significance of up-regulation of β cell Ca_V_3.1 channels in the pathogenesis of human diabetes.

Ca_V_3.1 channels exist primarily for mediating Ca^2+^ influx into the cytosolic compartment to participate in the formation of [Ca^2+^]_i_ dynamics that regulates glucose-stimulated insulin secretion and maintains β cell specificity (1, 5–7). This prompted us to examine whether Ad-EGFP-Ca_V_3.1 transduction altered [Ca^2+^]_i_ dynamics, leading to impaired glucose-stimulated insulin secretion. It turned out that both Ad-EGFP-Ca_V_3.1-transduced rat and human islets showed significant elevations in [Ca^2+^]_i_ during perifusion with 3 mM glucose and stimulation with 16.7 mM glucose or 25 mM KCl compared to those treated with Ad-EGFP (Fig. 1 *E* and *F*). [Ca^2+^]_i_ curves registered from Ad-EGFP-Ca_V_3.1-transduced islets were shifted upwards in comparison to those from islets transduced with Ad-EGFP (Fig. 1 *E* and *F*). These results substantiate the importance of the up-expressed Ca_V_3.1 channels in rearranging [Ca^2+^]_i_ dynamics and thereby elevation in [Ca^2+^]_i_ in islets. However, intuitively, such an elevation in [Ca^2+^]_i_ should promote insulin secretion by allosterically triggering Ca^2+^-dependent SNARE interactions (23–25). This suggests that elevated [Ca^2+^]_i_ in Ad-EGFP-Ca_V_3.1-transduced islets may switch on other mechanisms to impair insulin secretion. Previous studies indicate the possibility that the elevation in basal [Ca^2+^]_i_ following Ad-EGFP-Ca_V_3.1 transduction may activate CaN to dephosphorylate FoxO1, inducing its nuclear retention and down-regulation of some exocytotic proteins (17–19).

### Islets Transduced with Ad-EGFP-Ca_V_3.1 Lose Their Ability to Normalize Hyperglycemia in Streptozotocin-Induced Diabetic Rats.

**Fig. 2.**
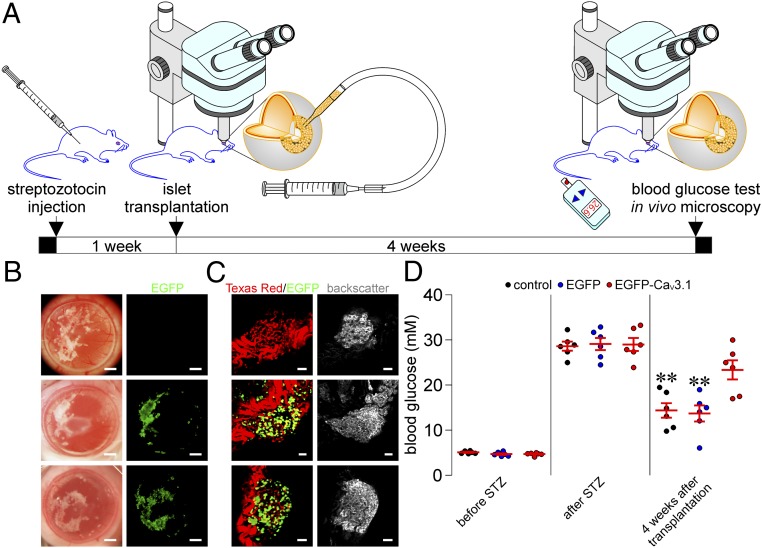
Effects of Ad-EGFP-Ca_V_3.1 transduction on antihyperglycemic ability of islets transplanted into the anterior chamber of the eye (ACE) of rats rendered diabetic with STZ injection. (*A*) Experimental protocol illustrating time points of STZ injection, islet transplantation, blood glucose test, and in vivo microscopy. (*B*) Stereomicroscopic photographs showing aggregated islets engrafted on the rat iris illuminated by visible light (*Left*) and EGFP fluorescence from these islet aggregates detected using the GFP filter set (*Right*). Control, Ad-EGFP-transduced, and Ad-EGFP-Ca_V_3.1-transduced islets are shown in the *Top*, *Middle*, and *Bottom* microphotographs, respectively. (Scale bars, 1 mm.) (*C*) Sample confocal images (*Left*) of control (*Top* microphotograph), Ad-EGFP-transduced (*Middle* microphotograph), and Ad-EGFP-Ca_V_3.1-transduced (*Bottom* microphotograph) islets engrafted on the rat iris. Green and red represent EGFP fluorescence from Ad-EGFP- and Ad-EGFP-Ca_V_3.1-transduced islets and Texas Red fluorescence from vasculatures filled with 70 kDa dextran-conjugated Texas Red, respectively. Corresponding reflected-light images (*Right*) of control (*Top* microphotograph), Ad-EGFP-transduced (*Middle* microphotograph), and Ad-EGFP-Ca_V_3.1-transduced (*Bottom* microphotograph) islets engrafted on the rat iris. (Scale bars, 50 μm.) (*D*) Quantifications of blood glucose levels in STZ-treated rats transplanted with control islets (*n* = 6) and islets transduced with Ad-EGFP (*n* = 6) or Ad-EGFP-Ca_V_3.1 (*n* = 6) before and after STZ injection and 4 wk after islet transplantation. **P < 0.01 vs. Ad-EGFP-Ca_V_3.1.

The impairment of first-phase glucose-stimulated insulin secretion represents the initial defect of β cell function and plays an important role in glucose intolerance during diabetes development (26–28). This prompted us to investigate the in vivo pathological role of Ad-EGFP-Ca_V_3.1-transduced islets that exhibited defective basal insulin release and first-phase glucose-stimulated insulin secretion. We compared the abilities of control, Ad-EGFP-infected, and Ad-EGFP-Ca_V_3.1-infected islets to ameliorate hyperglycemia in streptozotocin (STZ)-induced diabetic rats. These 3 groups of islets were transplanted into the anterior chamber of the eye of STZ-treated rats (Fig. 2*A*). All of them were well engrafted on the iris and richly vascularized within 4 wk after transplantation, during which both Ad-EGFP-Ca_V_3.1- and Ad-EGFP-treated islets emitted appreciable EGFP fluorescence (Fig. 2 *B* and *C*). STZ-treated rats manifested overt hyperglycemia before islet transplantation (Fig. 2*D*). Importantly, a significant normalization of hyperglycemia occurred in STZ-treated rats transplanted with either control islets or islets infected with Ad-EGFP but not in STZ-treated rats transplanted with Ad-EGFP-Ca_V_3.1-transduced islets (Fig. 2*D*). Indeed, Ad-EGFP-Ca_V_3.1-treated islets have no ability to ameliorate hyperglycemia in STZ-induced diabetic rats. Of note, no appreciable differences were found in the islet backscatter signal, which reflects functional islet cell mass (29), between control, Ad-EGFP-transduced, and Ad-EGFP-Ca_V_3.1-transduced islets (Fig. 2*C*). This indicates that the enhanced expression of β cell Ca_V_3.1 channels did not reach the extremely high level where these channels mediate exaggerated Ca^2+^ influx to cause Ca^2+^-dependent islet cell damage, such as reduced insulin content, apoptosis, and necrosis.

### Transduction with Ad-EGFP-Ca_V_3.1 Decreases Cytoplasmic p-FoxO1 and Induces FoxO1 Nuclear Retention through the Ca_V_3.1 Channel–Dependent Activation of Calcineurin.

**Fig. 3.**
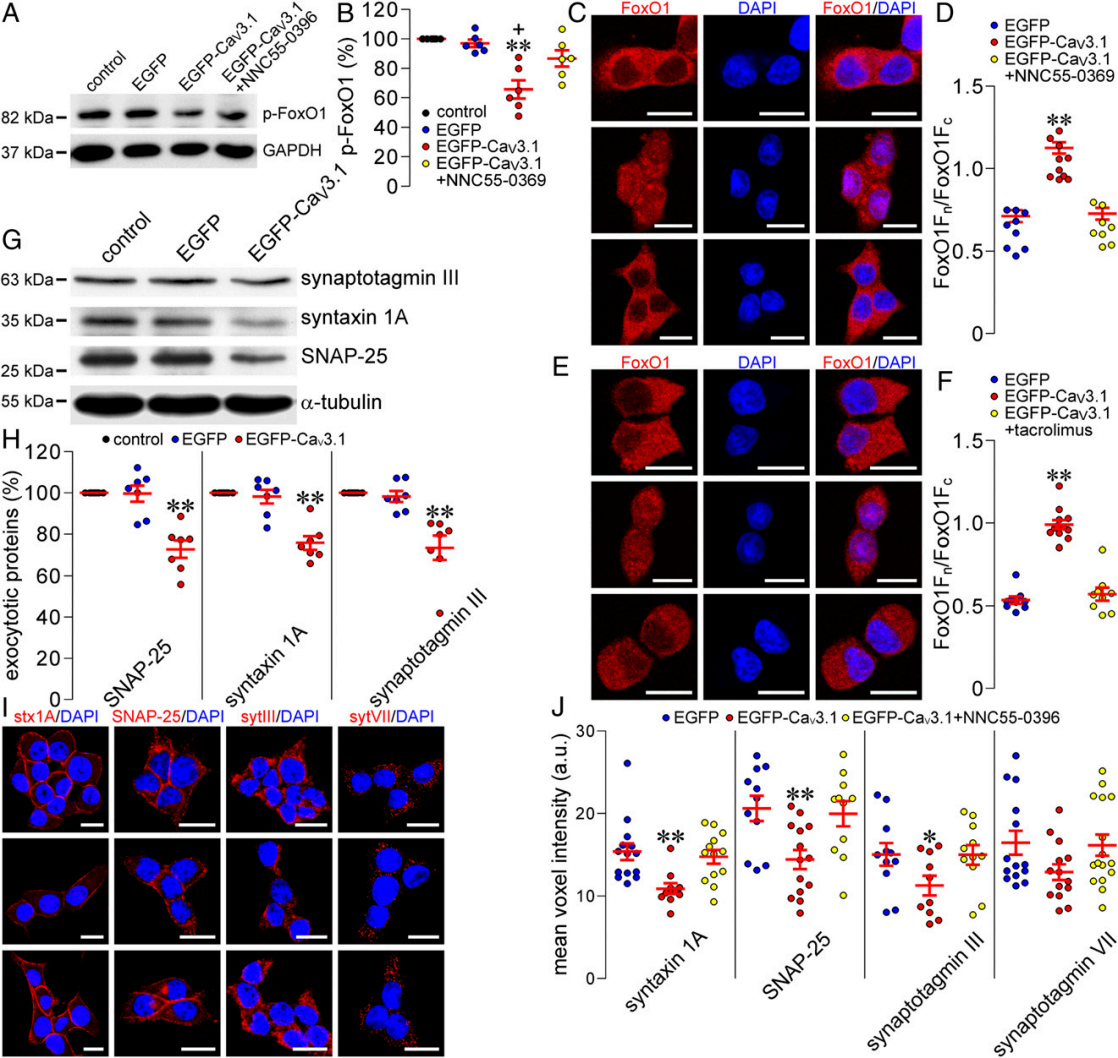
Effects of Ad-EGFP-Ca_V_3.1 transduction in combination with inhibition of Ca_V_3.1 channels or calcineurin on p-FoxO1, FoxO1, syntaxin 1A, SNAP-25, and synaptotagmin III and VII levels. (*A*) Representative blots of cytoplasmic p-FoxO1 and glyceraldehyde-3-phosphate dehydrogenase (GAPDH) bands in control INS-1E cells and cells transduced with Ad-EGFP, Ad-EGFP-Ca_V_3.1, or Ad-EGFP-Ca_V_3.1 in the presence of NNC55-0396. (*B*) Quantifications of p-FoxO1 immunoreactivity in the control group (*n* = 6) and groups transduced with Ad-EGFP (*n* = 6), Ad-EGFP-Ca_V_3.1 (*n* = 6), and Ad-EGFP-Ca_V_3.1 in the presence of NNC55-0396 (*n* = 6). **P < 0.01 vs. control and Ad-EGFP; ^+^*P* < 0.05 vs. Ad-EGFP-Ca_V_3.1 plus NNC55-0396. (*C*) Representative FoxO1 immunofluorescence (*Left* column), DAPI fluorescence (*Middle* column), and their overlay images (*Right* column) in Ad-EGFP-transduced INS-1E cells (*Upper* row) and Ad-EGFP-Ca_V_3.1-transduced INS-1E cells in the absence (*Middle* row) and presence (*Lower* row) of NNC55-0396. Red and blue represent FoxO1 immunofluorescence and DAPI fluorescence, respectively. (Scale bars, 10 μm.) (*D*) Quantification of FoxO1 immunofluorescence in the Ad-EGFP (*n* = 9), Ad-EGFP-Ca_V_3.1 (*n* = 10), and Ad-EGFP-Ca_V_3.1 + NNC55-0396 (*n* = 8) groups. **P < 0.01 vs. Ad-EGFP and Ad-EGFP-Ca_V_3.1 + NNC55-0396. FoxO1F_c_, cytoplasmic FoxO1 immunofluorescence. FoxO1F_n_, nuclear FoxO1 immunofluorescence. (*E*) Sample FoxO1 immunofluorescence (*Left* column), DAPI fluorescence (*Middle* column), and their overlay images (*Right* column) in Ad-EGFP-transduced INS-1E cells (*Upper* row) and Ad-EGFP-Ca_V_3.1-transduced INS-1E cells without (*Middle* row) and with (*Lower* row) exposure to tacrolimus. Red and blue represent FoxO1 immunofluorescence and DAPI fluorescence, respectively. (Scale bars, 10 μm.) (*F*) Quantitative analysis of FoxO1 immunofluorescence in the Ad-EGFP (*n* = 9), Ad-EGFP-Ca_V_3.1 (*n* = 12), and Ad-EGFP-Ca_V_3.1 + tacrolimus (*n* = 9) groups. **P < 0.01 vs. Ad-EGFP and Ad-EGFP-Ca_V_3.1 + tacrolimus. FoxO1F_c_, cytoplasmic FoxO1 immunofluorescence; FoxO1F_n_, nuclear FoxO1 immunofluorescence. (*G*) Representative blots of synaptotagmin III-, syntaxin 1A–, SNAP-25- and α-tubulin-immunoreactive bands in control islets and islets infected with Ad-EGFP or Ad-EGFP-Ca_V_3.1. (*H*) Quantifications of synaptotagmin III, syntaxin 1A, and SNAP-25 immunoreactivities in the control (*n* = 5 for SNAP-25, *n* = 7 for syntaxin 1A, and *n* = 7 for synaptotagmin III), Ad-EGFP (*n* = 5 for SNAP-25, *n* = 7 for syntaxin 1A, and *n* = 7 for synaptotagmin III), and Ad-EGFP-Ca_V_3.1 (*n* = 5 for SNAP-25, *n* = 7 for syntaxin 1A, and *n* = 7 for synaptotagmin III) groups. **P < 0.01 vs. control and Ad-EGFP. (*I*) Representative syntaxin 1A (stx1A, first column), SNAP-25 (second column), synaptotagmins III (sytIII, third column) and VII (sytVII, fourth column) immunofluorescence (red) overlaid with DAPI fluorescence (blue) in Ad-EGFP-transduced (*Upper* row) and Ad-EGFP-Ca_V_3.1-transduced INS-1E cells untreated (*Middle* row) and treated (*Lower* row) with NCC55-0396. (Scale bars, 10 μm.) (*J*) Quantitative analysis of syntaxin 1A, SNAP-25, and synaptotagmin III and VII immunofluorescence in the Ad-EGFP (*n* = 14 for syntaxin 1A, *n* = 11 for SNAP-25, *n* = 11 for synaptotagmin III, and *n* = 14 for synaptotagmin VII), Ad-EGFP-Ca_V_3.1 (*n* = 10 for syntaxin 1A, *n* = 14 for SNAP-25, *n* = 10 for synaptotagmin III, and *n* = 14 for synaptotagmin VII), and Ad-EGFP-Ca_V_3.1 + NNC55-0396 (*n* = 13 for syntaxin 1A, *n* = 11 for SNAP-25, *n* = 11 for synaptotagmin III, and *n* = 16 for synaptotagmin VII) groups. **P < 0.01 and *P < 0.05 vs. EGFP and Ad-EGFP-Ca_V_3.1 + NNC55-0396. a.u., arbitrary units.

The unique ability of β cells to accurately release insulin in response to glucose critically relies on adequate expression of β cell–specific genes under the control of a defined set of transcription factors, including FoxO1 (18, 30, 31). This transcription factor acts not only downstream of complex Ca^2+^ signaling systems but also upstream of expression of β cell exocytotic proteins (18, 31). This made us question if elevated expression of Ca_V_3.1 channels and resulting Ca^2+^ influx interfere with FoxO1 transcriptional action on β cell exocytotic protein genes with consequent impaired glucose-stimulated insulin secretion. We therefore quantified cytoplasmic phosphorylated FoxO1 in insulin-secreting INS-1E cells subjected to different treatments. It turned out that the relative abundance of cytoplasmic phosphorylated FoxO1 was significantly reduced in the Ad-EGFP-Ca_V_3.1 group in comparison to that in the control and the Ad-EGFP groups as well as the group subjected to Ad-EGFP-Ca_V_3.1 infection followed by exposure to the highly selective Ca_V_3 channel blocker NNC55-0396 (Fig. 3 *A* and *B*). All 4 groups exhibited similar intensities of GAPDH immunoreactivity (Fig. 3*A*). The reduction of cytoplasmic phosphorylated FoxO1 induced by elevated expression of Ca_V_3.1 channels reflects decreased phosphorylation and increased retention of FoxO1 in the nucleus and suggests that the expression of β cell exocytotic protein genes downstream of FoxO1 is reduced.

To detect up-expressed Ca_V_3.1 channel–induced changes in FoxO1 subcellular distribution and underlying mechanisms, we immunocytochemically characterized cytoplasmic and nuclear FoxO1 in INS-1E cells following Ad-EGFP-Ca_V_3.1 transduction and treatment with NNC55-0396 or the CaN inhibitor tacrolimus. Fig. 3 *C* and *E* shows that FoxO1 immunofluorescence was more intense in the nuclei but less intense in the cytoplasm of cells transduced with Ad-EGFP-Ca_V_3.1 than in those of Ad-EGFP-transduced cells. As shown in Fig. 3 *D* and *F*, the ratio of nuclear to cytoplasmic FoxO1 in the Ad-EGFP-Ca_V_3.1 group significantly increased compared to that in Ad-EGFP group. Importantly, the effects were effectively ablated by treatment with NNC55-0396 or tacrolimus (Fig. 3 *C*–*F*). These results demonstrate that Ad-EGFP-Ca_V_3.1 transduction indeed gives rise to FoxO1 nuclear retention through the Ca_V_3.1 channel–dependent activation of CaN. They also suggest that the up-expressed Ca_V_3.1-mediated Ca^2+^ influx is likely to constitutively hyperactivate CaN to make this phosphatase powerful enough to reach and dephosphorylate nuclear p-FoxO1, thereby preventing it from being extruded from the nucleus. In fact, the constitutively hyperactivated CaN has been demonstrated to dephosphorylate serine-256 of FoxO1 in ischemic neurons where [Ca^2+^]_i_ is pathologically high (17, 32).

### Ad-EGFP-Ca_V_3.1 Transduction Reduces Syntaxin 1A, SNAP-25, and Synaptotagmin III in a Ca_V_3.1 Channel–Dependent Manner.

To verify if FoxO1 nuclear retention induced by up-expressed Ca_V_3.1 channels reduces expression of β cell exocytotic protein genes, we performed immunoquantification of syntaxin 1A, SNAP-25, and synaptotagmins III and VII in rat islets and INS-1E cells. Immunoblot analysis shows that Ad-EGFP-Ca_V_3.1-treated islets gave significantly weaker intensities of synaptotagmin III-, syntaxin 1A-, and SNAP-25-immunoreactive bands in comparison to control and Ad-EGFP-treated islets (Fig. 3 *G* and *H*). These 3 types of islets exhibited similar intensities of α-tubulin-immunoreactive bands (Fig. 3*G*). Quantitative immunocytochemistry reveals that INS-1E cells transduced with Ad-EGFP-Ca_V_3.1 exhibited significant reduction in the immunofluorescence of syntaxin 1A, SNAP-25, and synaptotagmin III compared to Ad-EGFP-infected cells (Fig. 3 *I* and *J*). Interestingly, the reduction was efficiently reversed following NNC55-0396 treatment (Fig. 3 *I* and *J*). However, the critical Ca^2+^ sensor synaptotagmin VII that triggers insulin exocytosis only underwent marginal changes following the same treatments (Fig. 3 *I* and *J*). These results verify that FoxO1 nuclear retention resulting from Ad-EGFP-Ca_V_3.1 transduction suppresses transcription of syntaxin 1A, SNAP-25, and synaptotagmin III, which are critical for glucose-stimulated insulin secretion (6, 33–35). This is consistent with the fact that FoxO1 can bind to the promotor regions of SNARE genes and suppress their expression (19).

As a matter of fact, complex interactions between multiple genetic variants and numerous environmental factors result in a number of etiologically heterogeneous subgroups of diabetes (36). Some of them are characterized by derangements in a plethora of molecular and cellular events, including Ca_V_ channel–centered molecular networks in β cells (6, 37, 38). De novo up-expression of β cell Ca_V_3.1 channels plus pharmacological inhibition and characterization of the eventual ability of these genetically transduced islets to secrete insulin and normalize hyperglycemia enable us to dissect the role of elevated expression of β cell Ca_V_3.1 channels in the development of diabetes from the aforementioned complexity. Furthermore, the combination of such approaches with analysis of the FoxO1 signaling pathway allowed us to identify a pathway from the up-expressed β cell Ca_V_3.1 channels to transcriptional loci suppressing the expression of some exocytotic protein genes in the β cell. Limitations of the present work are due to the difficulty in obtaining islets from a large population of human donors with diabetes for experiments and recruiting sufficient numbers of patients with gain-of-function mutation of the Ca_V_3.1 gene or epigenetic up-regulation of Ca_V_3.1 gene expression for clinical trials. In this context, etiological heterogeneity in diabetes may hinder these basic and clinical studies since small sample sizes can bring about potential bias of experimental results (36). Our work suggests that enhanced expression of β cell Ca_V_3.1 channels plays a causal role in pathogenesis of human diabetes. This remains to be verified by detailed clinical studies.

## Conclusions

**Fig. 4.**
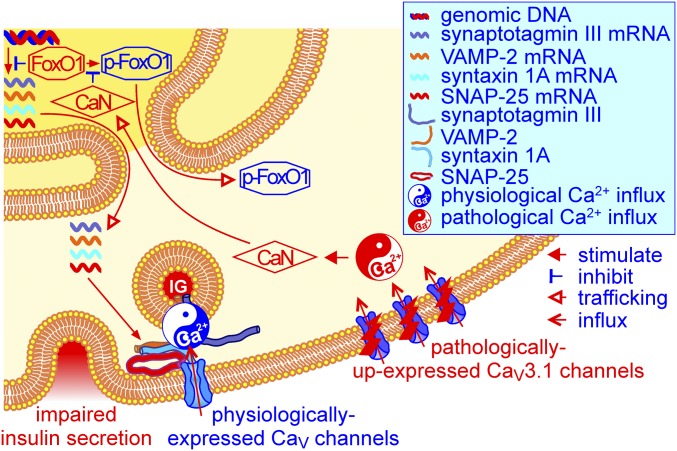
Model depicting how elevated expression of β cell Ca_V_3.1 channels impairs insulin release and glucose homeostasis. Unphosphorylated FoxO1 can bind to the promotor region of certain exocytotic protein genes to suppress their transcription. Under physiological conditions, however, the transcriptional suppression of FoxO1 hardly occurs since FoxO1 is phosphorylated and extruded from the nucleus to the cytoplasm. This ensures precise transcriptomics, correct differentiation, and adequate insulin secretion capacity of the β cell, thereby maintaining satisfactory glucose homeostasis in the body. Pathologically up-expressed Ca_V_3.1 channels mediate abnormal Ca^2+^ influx that constitutively hyperactivates CaN. Constitutively hyperactivated CaN dephosphorylates FoxO1 in nuclei, resulting in its nuclear accumulation. This leads to transcriptional suppression of the exocytotic proteins syntaxin 1A, SNAP-25, and synaptotagmin III, resulting in impaired insulin secretion and aberrant glucose homeostasis. IG, insulin granule.

We demonstrated the feasibility of using Ad-EGFP-Ca_V_3.1 to elevate Ca_V_3.1 channel expression in islets. The elevated expression of Ca_V_3.1 channels not only impairs both basal insulin release and first-phase glucose-stimulated insulin secretion with no influence on second-phase insulin response but also disables islets from normalizing hyperglycemia in STZ-induced diabetic rats. This happens since up-expressed Ca_V_3.1 channels mediate excessive Ca^2+^ influx, resulting in pathological elevation of basal [Ca^2+^]_i_, which sequentially brings about activation of CaN, dephosphorylation, and nuclear retention of FoxO1 and FoxO1-mediated suppression of syntaxin 1A, SNAP-25, and synaptotagmin III gene transcription in the β cell (Fig. 4). Enhanced T-type Ca^2+^ currents through β cell Ca_V_3.1 channels thus play a significant role in the development of a diabetic phenotype and suggest that β cell–specific blockade of these channels may be considered as an approach to treat diabetes.

## Methods

### Animals.

Specific pathogen-free Wistar rats at 8 to 12 wk of age were obtained from Charles River Laboratories (Sulzfeld, Germany) and maintained on a regular light–dark cycle (lights on at 0700 and off at 1900) in temperature- and humidity-controlled rooms and had free access to food pellets and tap water. All animal experiments were conducted according to the guidelines of the Animal Care Committee of Karolinska Institutet.

Additional experimental procedures are presented in the *SI Appendix*.

### Data Availability.

All of the data, associated protocols, and materials for this study are available within the paper and its *SI Appendix*.

## Supplementary Material

Supplementary File
