# Supplementary material for: Enhanced expression of β cell CaV3.1 channels impairs insulin release and glucose homeostasis
Source: Proc Natl Acad Sci U S A. 2019 Dec 23;117(1):448–53. doi: 10.1073/pnas.1908691117 (PMC6955371; doi:10.1073/pnas.1908691117)
Supplement: Supplementary File [file pnas.1908691117.sapp.pdf]

PNAS
www.pnas.org

Supplementary Information for

Enhanced expression of β cell $Ca_V3.1$ channels impairs insulin release and glucose homeostasis

Jia Yu, Yue Shi, Kaixuan Zhao, Guang Yang, Lina Yu, Yuxin Li, Eva-Marie Andersson, Carina Ämmälä, Shao-Nian Yang and Per-Olof Berggren

Jia Yu
E-mail: jia.yu@ki.se
Shao-Nian Yang
E-mail: shao-nian.yang@ki.se
Per-Olof Berggren
E-mail: per-olof.berggren@ki.se

This PDF file includes:

Supplementary text
SI Methods
Figures S1-S3
Table S1
SI References

www.pnas.org/cgi/doi/10.1073/pnas.1908691116

Supplementary Information Text

SI Methods

Pancreatic Islet Isolation. Rat islets were isolated using the *in situ* ductal perfusion technique. Rats were anesthetized with CO_2 and then killed by decapitation. Their abdomens were opened. About 15 ml of collagenase solution (1.2 mg/ml; Roche, Basel, Switzerland) was injected into the pancreas through the common bile duct. The distended pancreas was digested in collagenase solution for 20 minutes at 37°C. The digested pancreas was dissociated and rinsed with Hanks balanced salt solution (Invitrogen, Carlsbad, CA). The resultant islets were hand-picked and cultured in RPMI 1640 medium (Invitrogen) supplemented with 10% fetal bovine serum, 2 mM L-glutamine and 100 IU/100 µg/ml penicillin/streptomycin. Some of them were also dispersed into single islet cells. Both islets and dispersed islet cells were maintained in complete RPMI medium at 37°C in a humidified 5% CO_2 incubator.

Human islets were obtained from Prodo Laboratories Inc., providing islets isolated from donor pancreases obtained from deceased individuals with research consent from organ procurement organizations. The use and storage of human islets were performed in compliance with the Declaration of Helsinki, International Conference on Harmonisation/Good Clinical Practice, and AstraZeneca code of conduct. The human islets were hand-picked at their arrival, infected with Ad-EGFP and Ad-EGFP-Ca_V3.1 and maintained for 72 h in tissue culture in PIM(S) Complete (Prodo Laboratories Inc.) before use. Characteristics of human islet donors and purity of human islets are listed in *SI Appendix*, Table S1.

Cell Culture. INS-1E, COS-7 and HEK-293 cells at about 70% confluency were trypsinized. The trypsinized cells and dispersed rat islet cells were seeded onto the bottom of Petri dishes or glass coverslips. INS-1E cells were grown in RPMI 1640 medium containing the following supplements: 10% fetal bovine serum, 2 mM L-glutamine, 100 U/100 µg/ml penicillin/streptomycin, 10 mM HEPES, 1 mM sodium pyruvate and 50 µM β-mercaptoethanol (Invitrogen). COS-7 and HEK-293 cells were cultured in DMEM (Invitrogen) supplemented with 10% fetal bovine serum, 1 mM L-glutamine and 50 IU/50 µg/ml penicillin/streptomycin. The islet cells were cultivated in RPMI 1640 medium supplemented with 10% fetal bovine serum, 2 mM L-glutamine, and 100 IU/100 µg/ml penicillin/streptomycin (Invitrogen). After overnight cultivation at 37°C in a humidified 5% CO_2 incubator, cells were subjected to Ad-EGFP and Ad-EGFP-Ca_V3.1 infection.

Recombinant Adenovirus Preparation and Infection. Adenovirus type 2-derived replication-deficient recombinant vectors (Ad) carrying either EGFP or EGFP-Ca_V3.1 in the viral E1 region were constructed using the two-cosmid system (1, 2). The coding DNA sequence for the EGFP-Ca_V3.1 subunit was generated by fusing EGFP to the amino-terminal end of the Ca_V3.1 subunit. Coding DNA sequences for either EGFP or EGFP-Ca_V3.1 were subcloned into the adenoviral shuttle vector pLEPMV6, a pLEP derivative carrying the cytomegalovirus promoter and an SV40 poly(A) signal, to generate the corresponding genomic adenoviral cosmids. Transfection of HEK-293 cells with the resultant adenoviral cosmids was performed to produce Ad-EGFP and Ad-EGFP-Ca_V3.1. The correct DNA inserts were verified by polymerase chain reaction and restriction enzyme analyses. Ad-EGFP and Ad-EGFP-Ca_V3.1 were further amplified in HEK-293 cells and purified with Adeno-X™ Maxi Purification Kit (Clontech, Mountain View, CA). The obtained Ad-EGFP and Ad-EGFP-Ca_V3.1 vectors at 2-5 x 10^{2-4} adenovirus particles per cell were used to infect overnight-cultured COS-7, HEK-293, INS-1E and dispersed islet cells as well as isolated islets for 72 h at 37°C in a humidified 5% CO_2 incubator. The infected cells and islets were subjected to patch clamp experiments, confocal microscopy, insulin secretion assay, $[Ca^{2+}]_i$ measurements, immunoblot analysis and transplantation into the anterior chamber of the eye of rats.

Diabetes Induction, Islets Transplantation and Blood Glucose Monitoring. Diabetes in rats was induced by a single intraperitoneal injection of streptozotocin (STZ) (65 mg/kg; Sigma-Aldrich, St. Louis, MO). The STZ-treated rats manifested severe hyperglycemia and those with fasting blood glucose levels > 25 mM were subjected to islet transplantation at 1 week after STZ injection. In brief, control, Ad-EGFP- or Ad-EGFP-Ca_V3.1-treated islets were aspirated into a blunt 27-gauge eye cannula connected to a 1-ml Hamilton syringe. A recipient rat was anesthetized

with a mixture of 40% oxygen and 2.5% isoflurane. Its cornea was punctured with a sharp needle under a stereomicroscope. Subsequently, the blunt eye cannula was gently inserted through the punctured hole into the anterior chamber of the rat eye. Each STZ-treated rat was transplanted with 500 islets, equally divided between the two anterior chambers. Fasting blood glucose levels were monitored before and after STZ injection and after islet transplantation using an Accu-Chek™ blood glucose meter (Roche Diagnostics, Indianapolis, IN).

***In Vitro* and *In Vivo* Confocal Microscopy.** For *in vitro* confocal microscopy, Ad-EGFP- or Ad-EGFP-Ca_V3.1-infected islets were fixed in 4% paraformaldehyde and mounted in ProLong® Gold Antifade mountant with DAPI (Thermo Fisher, Waltham, MA) shortly before imaging. COS-7 cells infected with Ad-EGFP or Ad-EGFP-Ca_V3.1 were bathed in extracellular solution consisting of (in mM) 135 NaCl, 3.6 KCl, 5 $NaHCO_3$, 0.5 NaH_2PO_4, 0.5 $MgCl_2$, 1.5 $CaCl_2$, 10 HEPES and 0.1% bovine serum albumin. EGFP fluorescence was measured with a Leica TCS-SP2 or TCS SP8 X confocal laser-scanner connected to a Leica DMIRBE or DMi8 microscope (Leica Microsystems Heidelberg GmbH, Mannheim, Germany). EGFP and DAPI were excited by 488 nm and 405 nm laser lines and the resultant emissions were captured using Leica PL APO 63x/1.32 oil, PL APO 100x/1.40 oil or HC PL APO CS2 63x/1.30 GLYC objectives at 505-530 nm and 410-440 nm.

To perform *in vivo* confocal imaging, the rat was anesthetized with a mixture of 40% oxygen and 2.5% isoflurane at 4 weeks after islet transplantation and placed on a heating pad to keep its body temperature at 37°C. The head of the anesthetized rat was immobilized in a head holding adaptor (SG-3N; Narishige, Tokyo, Japan) that tilts the eye containing the engrafted islets to a proper orientation. For visualization of vascular structures, the rat was intravenously injected with 70 kDa dextran-conjugated Texas Red (Molecular Probes, Eugene, OR). GFP-positive islet cells and vascular structures filled with dextran-conjugated Texas Red within the engrafted islets were visualized under DMIRBE microscope equipped with a Leica TCS-SP2 confocal laser-scanner. GFP and Texas Red were excited by 488 nm and 595 nm laser lines and the resultant emissions were captured using a long-distance water-dipping lens (Leica HXC APO 10x/0.3W) at 505-530 nm and 610-630 nm, respectively. Reflection images of islets were obtained with 633 nm illumination and collection of light between 630 and 636 nm.

Electrophysiology. COS-7 cells and rat islet cells were subjected to single channel and whole-cell patch-clamp measurements following infection with Ad-EGFP and Ad-EGFP-Ca_V3.1. Cell-attached and conventional whole-cell patch-clamp configurations were employed. Single channel and whole-cell currents were recorded with an Axopatch 200B amplifier (Molecular Devices, Foster City, California) and an EPC-9 patch clamp amplifier (HEKA Elektronik, Lambrecht/Pfalz, Germany), respectively, at room temperature (about 22°C). Recording electrodes were made from borosilicate glass capillaries, fire-polished and coated with Sylgard close to their tips. Some of them were filled with a solution containing (in mM) 110 $BaCl_2$, 10 TEA-Cl, and 5 HEPES (pH 7.4 with $Ba(OH)_2$ for single channel measurements. Others were filled with a solution composed of (in mM) 150 N-methyl-D-glucamine, 125 HCl, 10 EGTA, 1.2 $MgCl_2$, 3 MgATP, and 5 HEPES (pH 7.15) for whole-cell current recordings. Electrode resistance ranged between 4 and 6 MΩ when they were filled with electrode solutions and immersed in bath solutions. The electrode offset potential was corrected in bath solutions prior to gigaseal formation. Single-channel recordings were performed with cells bathed in a depolarizing external recording solution, containing (in mM) 125 KCl, 30 KOH, 10 EGTA, 2 $CaCl_2$, 1 $MgCl_2$, and 5 HEPES-KOH (pH 7.15). This solution was used to bring the intracellular potential to 0 mV. For whole-cell current measurements, the cells were bathed in a solution containing (in mM) 148 tris(hydroxymethyl)aminomethane, 5.6 KCl, 1.2 $MgCl_2$, 10 $CaCl_2$ and 5 HEPES (pH 7.4). Acquisition and analysis of single channel and whole-cell current data were done using the software program pCLAMP 10 (Axon Instruments) and the software program PatchMaster/FitMaster (HEKA), respectively.

$[Ca^{2+}]_i$ Measurements. Ad-EGFP- or Ad-EGFP-Ca_V3.1-infected rat and human islets were loaded with 2 μM fura-2 LeakRes/AM for 60 min at 37°C in HEPES-buffered solution containing (in mM) 125 NaCl, 5.9 KCl, 2.56 $CaCl_2$, 1.2 $MgCl_2$, 25 HEPES, 3 glucose and 0.1% bovine serum albumin (pH 7.4). Subsequently, fura-2-loaded islets were immobilized onto a glass coverslip at

the bottom of a recording chamber and subjected to $[Ca^{2+}]_i$ measurements using a Spex Fluorolog spectrophotometer coupled to a Zeiss Axiovert 35 M microscope with a Zeiss Fluar 40×/1.30 oil objective (Carl Zeiss, Göttingen, Germany). The Fura-2 F340/F380 ratio was recorded to represent $[Ca^{2+}]_i$. Islets were purifused with HEPES-buffered solution supplemented with 3 mM, 16.7 mM glucose or 25 mM KCl at 37°C during recordings. Data were analyzed using Evtra software.

SDS-PAGE and Immunoblot Analysis. INS-1E cells and rat islets were lysed in a lysis buffer (pH 7.5) consisting of (in mM) 50 HEPES, 150 NaCl, 1 EGTA, 1 EDTA, 10% glycerol, 1% triton X-100, 1 PMSF and a protease inhibitor cocktail (Roche Diagnostics, Mannheim, Germany) following different treatments. The lysates were centrifuged at 800 X *g* for 10 min at 4°C to remove cell debris and nuclei. The protein concentration of the resulting samples was determined with Bio-Rad protein assay reagent (Bio-Rad, Hercules, CA). The lysates were denatured by heating at 96°C for 4 min in SDS sample buffer and subjected to sodium dodecyl sulfate-polyacrylamide gel electrophoresis (SDS-PAGE) and immunoblot analysis. 60 µg of proteins were separated in discontinuous gels consisting of a 4% acrylamide stacking gel (pH 6.8) and an 10% acrylamide separating gel (pH 8.8). The separated proteins were then electroblotted to hydrophobic polyvinylidene difluoride membrane (Hybond-P; GE Healthcare, Uppsala, Sweden). The blots were blocked by incubation for 1 h with 5% non-fat milk powder in a washing buffer, containing (in mM) 20 tris(hydroxymethyl)aminomethane, 150 NaCl and 0.05% Tween 20 (pH 7.5). They were then incubated overnight at 4ºC with rabbit polyclonal antibodies to synaptotagmin III (1:1000; Catalog number: ab81538, Abcam, Cambridge, UK) and phospho-FoxO1 (Ser256) (1:1000; Catalog number: 9461, Cell Signaling Technology, Danvers, MA), rabbit monoclonal antibody to GAPDH (1:1000; Catalog number: 2118, Cell Signaling Technology), and mouse monoclonal antibodies to syntaxin 1A (1:2000; Catalog number: S0664, Sigma-Aldrich, St. Louis, MO), SNAP-25 (1:1000; Catalog number: SMI-81R, Covance, Emeryville, CA) and α-tubulin (1:600; Catalog number: sc-8035, Santa Cruz Biotechnology, Santa Cruz, CA), respectively. After rinsing with the washing buffer, the blots were incubated with the secondary antibodies horseradish peroxidase-conjugated goat anti-rabbit IgG or horseradish peroxidase-conjugated goat anti-mouse IgG (1:50,000; Bio-Rad, Hercules, CA) at room temperature for 45 min. The immunoreactive bands were visualized with the Immun-Star™ HRP chemiluminescent kit (Bio-Rad).

Immunofluorescence Labeling and Quantitative Confocal Imaging. INS-1E cells were subjected to Ad-EGFP transduction or transduction with Ad-EGFP-Ca_V3.1 in the absence and presence of NNC55-0396 (1 µM, Tocris, Minneapolis, MN) or tacrolimus (30 ng/ml, Sigma-Aldrich). After treatments, cells adherent to glass coverslips were fixed in 4% paraformaldehyde for 20 min or 100% methanol for 5 min and then incubated with rabbit monoclonal anti-FoxO1 (1:100; Catalogue number: 2880, Cell Signaling, Leiden, Netherlands), rabbit polyclonal anti-syntaxin 1A (1:100; Catalogue number: ab41453, Abcam, Cambridge, MA), mouse monoclonal anti-SNAP-25 (1:1000; Catalogue number: 111011, Synaptic Systems, Göttingen, Germany), rabbit polyclonal anti-synaptotagmin III (1:500; Catalogue number: 105133, Synaptic Systems, Göttingen, Germany) and rabbit polyclonal anti-synaptotagmin VII antibodies (1:200; Catalogue number: 105173, Synaptic Systems, Göttingen, Germany), respectively, at 4ºC overnight or room temperature for 2 h. The specimens were thereafter treated with goat anti-rabbit IgG or anti-mouse IgG1 coupled to Alexa 633 or 546 (1:1000, Thermo Fisher, Waltham, MA) at room temperature for 1 h. After antibody labeling, specimens were mounted in ProLong® Gold Antifade mountant with DAPI (Thermo Fisher, Waltham, MA) to prevent photobleaching of fluorophores conjugated to secondary antibodies and to counterstain cell nuclei. Omission of the primary antibodies, incubation with nonimmune IgG from corresponding species or preabsorption of antigen peptides were used as controls. The dilution and incubation period of primary and secondary antibodies and other procedures were kept consistent among batches of experiments. The labeled cells were visualized with a Leica TCS SP8 X confocal laser scanner equipped with a white light and 405 nm pulsed laser and connected to a Leica DMi8 microscope. Alexa 633, Alexa 546 and DAPI were excited by 633 nm, 546 nm and 405 nm laser lines, respectively, and the resultant emissions were captured using a Leica PL APO CS2 63x/1.30 GLYC objective at 645-

730 nm, 560-630 nm and 410-440 nm, respectively. The same parameters and settings were used for imaging the labeled cells from batch to batch. Confocal images and their voxel intensities were analyzed with Volocity software (PerkinElmer). The ratio of the mean voxel intensity of nuclear FoxO1 immunofluorescence image to that of cytoplasmic FoxO1 immunofluorescence image was used as the index of FoxO1 nuclear retention and the mean voxel intensity of syntaxin 1A, SNAP-25, synaptotagmin III and VII immunofluorescence images was used as the readout of expression levels of these proteins.

Islet Perifusion and Insulin Secretion Assay. Groups of 50 isolated rat islets untreated or infected with Ad-EGFP or Ad-EGFP-$Ca_V3.1$ were transferred to 0.27-ml columns containing Bio-Gel P4 polyacrylamide beads (Bio-Rad, Hercules, CA) and perifused at a flow rate of 150 µl/min at 37°C. Effluent perifusate samples were collected every min for the duration of the experiments. Perifusion solution consisted of (in mM) 119 NaCl, 4.6 KCl, 2 $CaCl_2$, 1 $MgSO_4$, 0.15 Na_2HPO_4, 0.4 KH_2PO_4, 5 $NaHCO_3$ and 20 HEPES (pH 7.4) as well as 0.1% bovine serum albumin supplemented with 3 mM or 16.7 mM glucose. Insulin concentrations in the collected samples were determined by using separation-free ArcDia™ TPX assay technique and normalized to islet DNA contents by using DNA quantification kit (Sigma-Aldrich).

Data Analysis. All data are presented as mean ± SEM. One-way ANOVA followed by least significant difference (LSD) test and Student's t test were used to detect statistically significant differences between multiple treatments and between two treatments, respectively. The significance level was set to < 0.05.

SI Figures

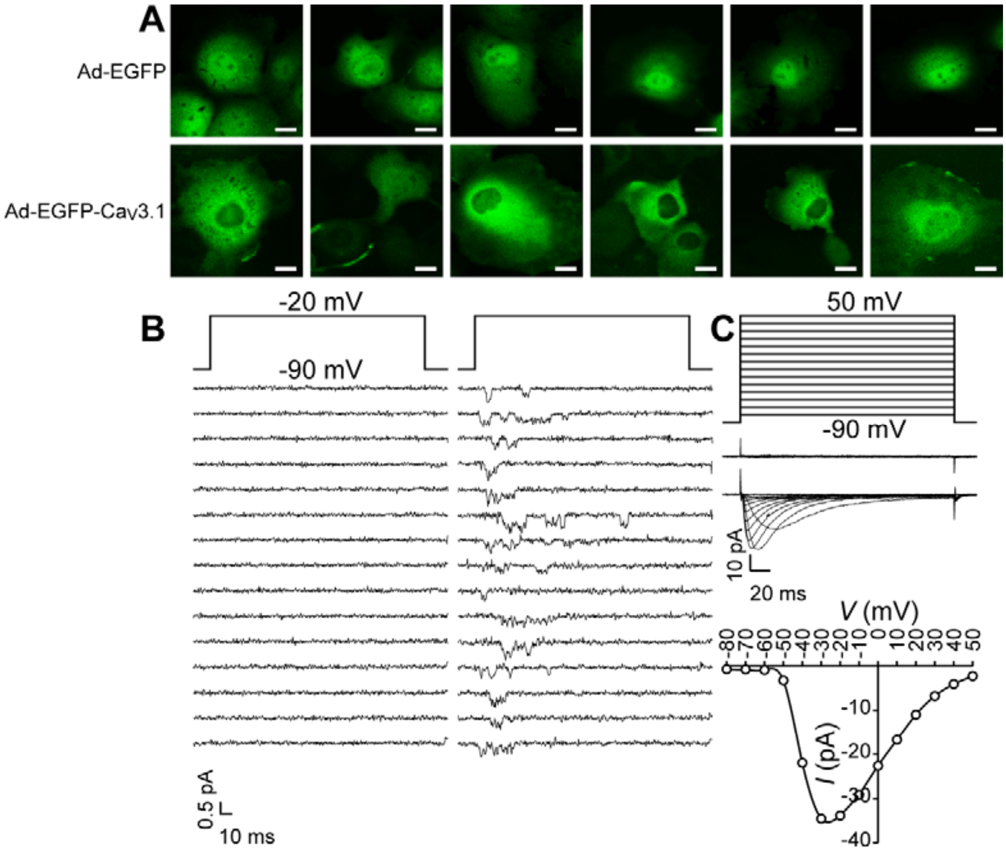

Fig. S1. Confocal microscopic and electrophysiological characterization of Ad-EGFP-Ca_V3.1-transduced COS-7. (*A*) Sample confocal images of Ad-EGFP- (top panel) and Ad-EGFP-Ca_V3.1-transduced COS-7 cells (bottom panel). Scale bars = 20 μm. (*B*) Sample unitary Ba^{2+} current traces from a cell infected with Ad-EGFP (left panel) and an Ad-EGFP-Ca_V3.1-transduced cell (right panel), respectively. (*C*) Sample whole-cell Ca_V current traces from a cell infected with Ad-EGFP (second panel) and an Ad-EGFP-Ca_V3.1-transduced cell (third and fourth panels), respectively.

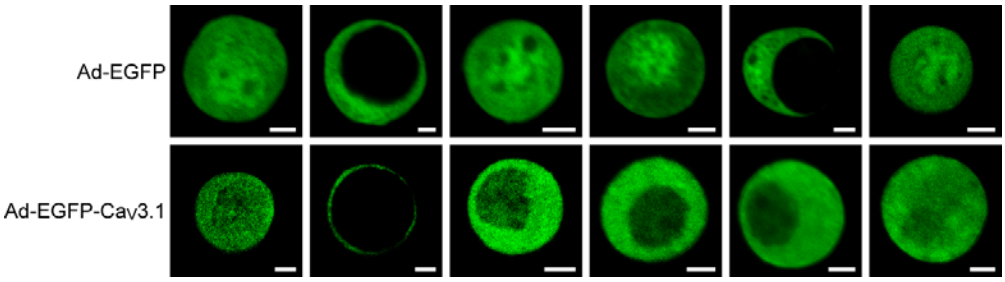

Fig. S2. Confocal images of Ad-EGFP- (top panel) and Ad-EGFP-Ca_V3.1-transduced rat islet cells (bottom panel). Scale bars = 3 μm.

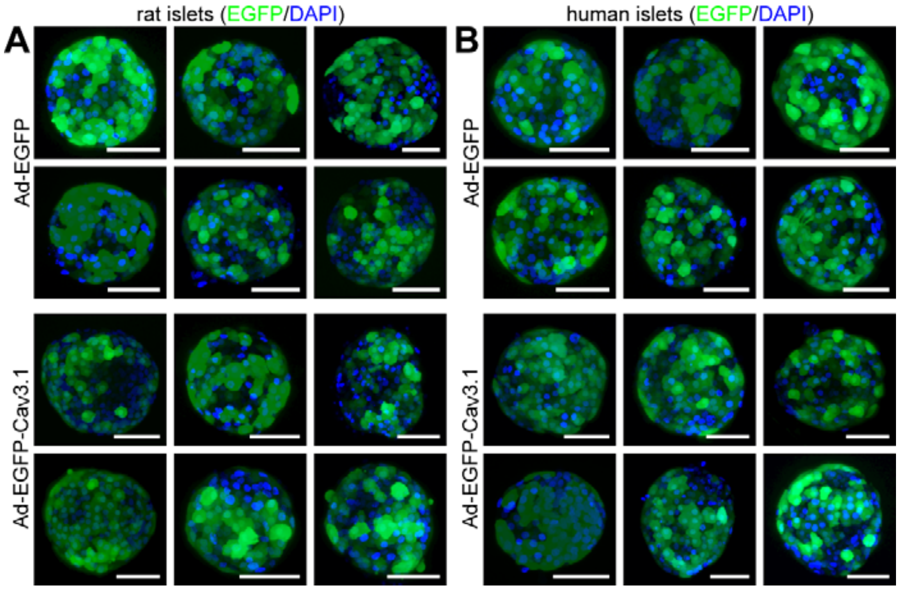

Fig. S3. Ad-EGFP- and Ad-EGFP-Ca$_V$3.1-transduced rat and human islets. (*A* and *B*) Sample confocal images of Ad-EGFP- (top two panels) and Ad-EGFP-Ca$_V$3.1-transduced (bottom two panels) rat (*A*) and human islets (*B*). Green and blue representing EGFP and DAPI fluorescence, respectively. Scale bars = 50 µm.

Table S1. Characteristics of human islet donors and purity of human islets

Subject's Number	Age	Sex	BMI	HbA1c	Cause of Death	Islet Purity
1	46	Male	27.6	5.4%	Head trauma	90%
2	38	Male	23.2	5.6%	Stroke	90%
3	18	Female	30.4	5.1%	Head trauma	85%
4	23	Male	25.1	5.6%	Head trauma	90%
5	35	Male	31.5	4.9%	Head trauma	90-95%
6	40	Male	25.0	5.3%	Head trauma	90-95%
7	29	Male	22.8	5.5%	Head trauma	85%
8	41	Male	23.5	5.2%	An anoxic event	95%
9	64	Male	25.5	5.4%	Stroke	90-95%
10	37	Male	23.9	5.8%	Head trauma	90%
11	38	Male	27.7	5.4%	Head trauma	85%
12	43	Female	31.7	5.7%	An anoxic event	95%
13	51	Female	24.8	5.2%	Stroke	95%
14	62	Male	31.2	5.5%	Stroke	90-95%

BMI: basic metabolic index.

References

1. X. Wang, W. Zeng, M. Murakawa, M. W. Freeman, B. Seed, Episomal segregation of the adenovirus enhancer sequence by conditional genome rearrangement abrogates late viral gene expression. *J. Virol.* **74**, 11296-11303 (2000).
2. P. O. Berggren *et al.*, Removal of Ca^{2+} channel β3 subunit enhances Ca^{2+} oscillation frequency and insulin exocytosis. *Cell* **119**, 273-284 (2004).
